# Performance of maize tassel activated carbon for COD removal from industrial wastewater under optimized conditions

**DOI:** 10.1038/s41598-025-24902-y

**Published:** 2025-11-18

**Authors:** Ghada A. El Gamal, Ahmed M. Gomaa

**Affiliations:** 1 Mechatronics Engineering Department, Faculty of Engineering and Technology, Egyptian Chinese University, Cairo, Egypt; 2 Construction and Building Engineering Department, Faculty of Engineering and Technology, Egyptian Chinese University, Cairo, Egypt

**Keywords:** Activated carbon, Industrial wastewater treatment, Contaminant removal, Adsorption technology, COD reduction, Engineering, Chemical engineering, Civil engineering, Mechanical engineering

## Abstract

Industrial wastewater, laden with hazardous organic and inorganic contaminants, poses critical environmental risks. Adsorption via activated carbon stands out as a scalable and efficient remediation strategy due to its simplicity and adaptability. This study evaluates maize tassel–derived activated carbon (MTAC), synthesized from agricultural waste, as a low-cost and sustainable solution to remove organic pollutants, with emphasis on reducing chemical oxygen demand (COD) in industrial effluents. A comprehensive assessment of critical parameters (pH, temperature, adsorbent dosage, contact time, and agitation speed) was performed to optimize the adsorption process. MTAC exhibited a high surface area of 510.4 m^2^/g, and characterization through FTIR, SEM, and XRD confirmed its porous structure and abundant surface functional groups. The adsorption performance was strongly influenced by pH, with the highest adsorption capacity (464 mg/g) and COD removal of 92.8% at pH 6 and 30 °C. Maximum efficiency (96.6%) was obtained at a dose of 3 g/L, with peak adsorption capacity (484 mg/g) achieved at 45 min and 200 rpm. Adsorption equilibrium data fitted well to the Langmuir model (R^2^ = 0.999), indicating monolayer adsorption with a maximum capacity of 484 mg/g, while Freundlich parameters confirmed favorable multilayer adsorption. Thermodynamic analysis revealed negative ΔG° values, confirming spontaneity, along with a negative ΔH° (− 0.523 kJ·mol⁻^1^), validating the exothermic nature of the process. Reusability tests demonstrated that MTAC retained more than 85% of its efficiency after five regeneration cycles, confirming its economic and practical viability. Comparative analysis further showed that MTAC outperformed commercial activated carbon (CAC) in adsorption capacity, COD removal, and cost-effectiveness. The utilization of maize tassel, an abundant agricultural byproduct, highlights its potential as a sustainable adsorbent that supports circular economy strategies and advances progress toward SDGs 6, 12, and 13.

**Supplementary Information:**

The online version contains supplementary material available at 10.1038/s41598-025-24902-y.

## Introduction

Recent decades of accelerated industrial growth and urban development have significantly increased both the volume and complexity of wastewater released into water bodies. This industrial effluent commonly contains a diverse array of harmful pollutants, such as heavy metals, synthetic dyes, phenolic compounds, hydrocarbons, and pharmaceutical contaminants^1–5^. These contaminants are frequently toxic, resistant to degradation, and chemically stable, which allows them to persist in the environment and withstand traditional treatment methods^6–10^. If not properly treated, these effluents can lead to severe environmental and public health risks, including bioaccumulation in the food chain, eutrophication of water bodies, carcinogenicity, mutagenicity, and endocrine disruption in both humans and wildlife^11–13^. The growing pressure on industries to comply with increasingly stringent wastewater discharge regulations has highlighted the need for more effective, cost-efficient, and sustainable treatment technologies^14,15^.

Conventional wastewater treatment techniques including coagulation-flocculation, chemical precipitation, ion exchange, membrane filtration, ozonation, electrochemical methods, and advanced oxidation processes have been extensively used to eliminate a variety of pollutants ^16–19^. Although these techniques are effective, they typically involve high operational expenses, sophisticated infrastructure, trained personnel, and produce secondary waste like sludge, adding further challenges to waste management^20,21^. In light of these challenges, industries are seeking more sustainable, economically viable, and environmentally friendly alternatives for wastewater treatment^22–24^.

One such promising technique is adsorption, which has gained widespread recognition as a highly effective and simple method for the removal of contaminants from aqueous solutions^25,26^. Adsorption is a non-destructive, scalable, and energy-efficient process that does not produce harmful by-products, making it ideal for industrial applications aimed at minimizing environmental impact^27–29^. Over the past two decades, adsorption using activated carbon (AC) has been extensively studied and applied for industrial wastewater treatment due to its remarkable physicochemical properties, including a large surface area, high porosity, and the presence of abundant surface functional groups that facilitate pollutant removal^30,31^.

Among the various forms of activated carbon, commercial activated carbon (CAC) has been the most widely used material for wastewater treatment. CAC is produced from carbonaceous materials such as coal, wood, and coconut shells and is characterized by its high surface area (ranging from 400 to over 1000 m^2^/g) and strong adsorption affinity for a variety of contaminants. Studies have shown that CAC can effectively adsorb organic pollutants such as dyes, phenols, and pharmaceuticals, as well as inorganic pollutants such as heavy metals including lead, cadmium, and chromium, often with adsorption capacities exceeding 200 mg/g under optimal conditions^32–34^.The effectiveness of CAC is largely attributed to its heterogeneous pore structure, surface charge properties, and chemical compatibility with a wide range of contaminants^11,35–38^.

Despite its widespread use, the high cost of commercial activated carbon remains a limiting factor for many industries, particularly in developing countries. As a result, there has been growing interest in alternative, low-cost adsorbents derived from agricultural residues and industrial by-products. Among these, maize tassels, an abundant agricultural waste product, have gained attention as a promising feedstock for producing activated carbon^39–41^. Maize tassel-based activated carbon (MTAC) offers several advantages, including its low cost, availability, and sustainability, as well as its potential for waste valorisation. Maize tassels are rich in cellulose and lignin, which are ideal for the production of activated carbon with high porosity and surface area^11,42–44^.

Several studies have explored the use of MTAC for the treatment of industrial wastewater, particularly for the removal of heavy metals and organic contaminants. For instance, Chaudhary et al. (2020) demonstrated that MTAC has high adsorption capacity for lead and cadmium, with maximum adsorption capacities reaching 150 mg/g for lead and 120 mg/g for cadmium under optimal conditions^45^. Similarly, Patel et al. (2019) investigated the potential of MTAC for the removal of methylene blue dye from aqueous solutions and found that it exhibited excellent removal efficiency, especially under acidic conditions^46^. Other studies have highlighted the effectiveness of MTAC in removing pharmaceutical residues, including ibuprofen and paracetamol, further supporting its versatility as an adsorbent material for a wide range of industrial pollutant^47^.

The adsorption performance of MTAC is influenced by several key process parameters, including pH, temperature, adsorbent dosage, contact time, and agitation speed. The effect of these parameters has been widely studied in the literature, with several investigations indicating that the adsorption efficiency of MTAC increases under acidic conditions due to enhanced electrostatic interactions between the adsorbent and the pollutants^48,49^. Temperature also plays a critical role in the adsorption process, with higher temperatures generally enhancing the mobility of contaminant molecules and improving their diffusion into the adsorbent’s pores. The adsorbent dosage, contact time, and agitation speed further influence the efficiency of the adsorption process, with optimal conditions varying depending on the type of contaminant and the nature of the wastewater^48,49^.

The characterization of MTAC is crucial for understanding its adsorption behaviour and optimizing the treatment process. Techniques such as FTIR (Fourier Transform Infrared Spectroscopy), SEM (Scanning Electron Microscopy), and XRD (X-ray Diffraction) are commonly used to examine the surface functional groups, morphology, and crystalline structure of activated carbon. FTIR analysis provides valuable information about the functional groups present on the surface of MTAC, which are responsible for the adsorption of contaminants. SEM and XRD, on the other hand, help to assess the pore structure and crystallinity of the adsorbent, which are essential factors that influence its adsorption capacity^50–52^.

In addition to its high adsorption capacity and performance, MTAC offers the added benefit of being derived from renewable, low-cost materials, making it a sustainable alternative to traditional activated carbon. The use of agricultural waste such as maize tassels not only reduces dependence on fossil fuels and other non-renewable resources but also contributes to waste management and the circular economy by turning agricultural by-products into valuable adsorbent materials^53–55^. This aligns with the growing global emphasis on sustainable development and resource conservation, particularly within the context of wastewater treatment.

This study aims to evaluate the adsorption performance of maize tassel-based activated carbon (MTAC) for the treatment of industrial wastewater under varying physicochemical conditions. The experimental parameters investigated include pH (2–8), temperature (30–60 °C), adsorbent dosage (0.01–0.1 g/L), contact time (10–60 min), and agitation speed (300–600 rpm). The findings will provide insights into the optimal conditions for pollutant removal using MTAC, offering a cost-effective and sustainable solution for industrial wastewater treatment. This research also contributes to achieving the Sustainable Development Goals (SDGs), particularly SDG 6 (Clean Water and Sanitation), SDG 12 (Responsible Consumption and Production), and SDG 13 (Climate Action), by promoting eco-friendly, economically viable technologies that support environmental sustainability.

Industrial wastewater encompasses a wide spectrum of pollutants depending on the source industry. Among these, the juice processing industry generates large volumes of wastewater characterized by high organic loads, primarily from fruit residues, sugars, and organic acids^4,9^. These effluents typically exhibit elevated COD and BOD levels, low pH values due to natural acids (e.g., citric acid), and high turbidity from suspended solids and pulp. If untreated, such discharges can severely affect aquatic ecosystems by depleting dissolved oxygen and promoting eutrophication. Due to the biodegradable nature of these pollutants, adsorption-based treatment using bio-derived activated carbon offers a promising solution for reducing environmental impacts while aligning with principles of circular economy and waste valorization^1–3^. In this study, wastewater was collected from a local juice processing factory in Cairo, Egypt, and used to evaluate the adsorption performance of maize tassel–derived activated carbon (MTAC). The choice of this effluent allows the assessment of MTAC in treating real-world, moderately contaminated industrial wastewater with significant organic content.

Numerous studies have explored biomass-derived activated carbon as an effective and sustainable adsorbent for wastewater treatment. However, maize tassel, a widely available agricultural byproduct, remains underutilized in this context. Compared to other well-studied precursors like coconut shell, rice husk, and bamboo, maize tassel has not been systematically evaluated for its structural advantages, adsorption efficiency, or economic potential. This study aims to fill this gap by synthesizing maize tassel–based activated carbon (MTAC) and assessing its performance in removing chemical oxygen demand (COD) from industrial wastewater. We emphasize both comprehensive material characterization and comparative benchmarking against commercial activated carbon (CAC). In doing so, the work contributes a new perspective on sustainable adsorbent development and supports circular economy goals through agricultural waste valorisation.

## Material and methods

### Preparation of the maize tassel-based activated carbon

**Fig. 1:**
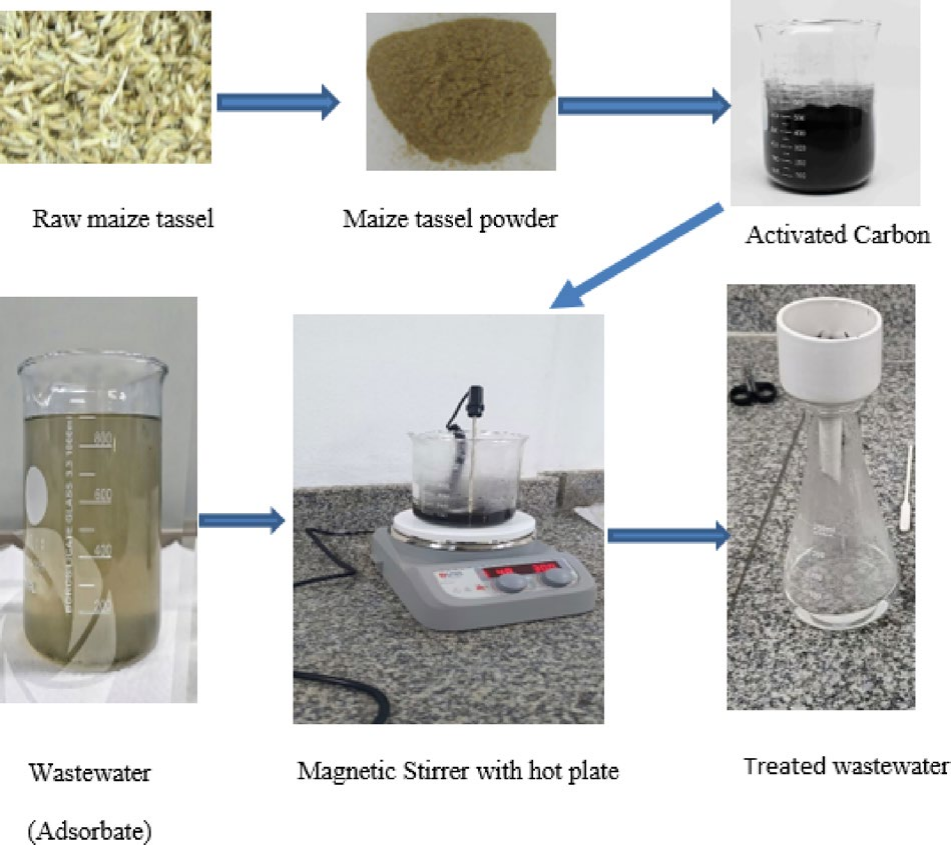
Schematic of MTAC synthesis and wastewater treatment process.

The synthesis of maize tassel-based activated carbon (MTAC) was performed through a two-step procedure: carbonization followed by chemical activation. Raw maize tassels were sourced from a local market in Cairo, Egypt. The biomass was thoroughly washed with distilled water to remove surface impurities, oven-dried at 105 °C for 24 h, and mechanically ground into a fine powder, as illustrated in Fig. 1.

The carbonization step was carried out in a tubular furnace under a nitrogen atmosphere at 500 °C for 2 h, yielding carbon-rich char. This char was then impregnated with phosphoric acid (H_3_PO_4_, 85%, analytical grade, Sigma-Aldrich, Germany) at a 1:1 weight ratio and left to soak for 24 h at ambient temperature. After impregnation, the material was dried at 100 °C, followed by a second thermal activation at 800 °C for 1.5 h under nitrogen to enhance porosity and develop surface functionality.

The activated product was washed several times with distilled water until the pH of the filtrate was neutral, indicating the removal of residual activating agent. The cleaned MTAC was then dried at 110 °C and stored in airtight containers for further use.

The synthesized MTAC was tested in a batch adsorption system for removing organic pollutants (primarily COD) from industrial wastewater collected from a juice processing factory located in Cairo, Egypt. A magnetic stirrer with integrated heating ensured uniform mixing throughout the adsorption process. After the optimum contact time, the treated samples were filtered, and the COD reduction was measured according to APHA (2017) Standard Methods. The sources and specifications of all materials used in the synthesis and experimental process are summarized in Table 1.

**Table 1:** Source and supplier of key materials used in MTAC synthesis and adsorption tests.

No	Material	Source/supplier	Description/purity
1	Maize tassel	Local market, Cairo, Egypt	Raw biomass
2	Phosphoric acid (H_3_PO_4_)	Sigma-Aldrich, Germany	85%, analytical grade
3	Industrial wastewater	Juice processing factory, Cairo, Egypt	Raw untreated effluent
4	Distilled water	Laboratory supply	For washing and dilution

### Optimization of the adsorption parameters

This study utilized maize tassel-derived activated carbon (MTAC) to mitigate contaminants and lower chemical oxygen demand (COD) in industrial wastewater. The adsorption efficacy was systematically evaluated across five operational variables: pH levels, contact time, adsorbent quantity, temperature, and agitation speed.

#### Batch adsorption experiments

All experiments in this study were conducted in batch mode at ambient room temperature to maintain procedural simplicity, reproducibility, and ease of operation. Batch mode was selected for its suitability in preliminary adsorption studies and its ability to control experimental variables precisely. The adsorption experiments were conducted in 250-mL borosilicate Erlenmeyer flasks, each containing a predetermined volume of industrial wastewater obtained from the Rockat fertilizer factory in New Salhia, Sharqia, Egypt (as detailed in Table 1). Activated carbon derived from maize tassels (MTAC), synthesized through a dual-stage protocol of carbonization and chemical activation, was employed as the adsorbent in all experimental trials.

Prior to each trial, a predetermined quantity of MTAC was measured and introduced into designated flasks. These flasks were then mounted on an orbital shaker operating at controlled rotational speeds (300–600 rpm) to ensure uniform dispersion and consistent interaction between the adsorbent and contaminants. The pH of wastewater samples was calibrated to target values (2–8) using diluted NaOH or HCl solutions, with adjustments validated via a calibrated pH meter before commencing each experiment.

Following pH adjustment, a measured volume of wastewater was introduced to the MTAC-containing flasks, and adsorption was initiated via continuous agitation. At specified intervals (10–60 min), flasks were sequentially retrieved from the shaker. The mixtures underwent filtration using Whatman No. 44 filter paper to isolate spent adsorbent from treated effluent. The filtrate was then analyzed to quantify residual contaminant concentrations, enabling evaluation of adsorption efficiency across varying operational conditions: contact duration (10–60 min), adsorbent dosage (0.01–0.1 g/L), temperature (30–60°C), and agitation intensity (300–600 rpm). All trials were replicated to validate reproducibility, with mean values applied for data interpretation. The following equations were applied to calculate the Chemical Oxygen Demand (COD) reduction percentage (%) and the equilibrium adsorption capacity.

1 $$\%COD \, Reduction=({COD}_{O}-{COD}_{e})/ {COD}_{O}\times 100$$

2 $${Q}_{e}=({COD}_{O}-{COD}_{e})\times V/W$$

For example, the initial and equilibrium COD concentrations (mg/L), are shown as CODo and CODe (ppm). The mass of the dried adsorbent is denoted as W (g), and V (mL) corresponds to the volume of contaminant-laden solution. Qe (mg/g) represents the equilibrium adsorption capacity of the adsorbent material.

### Testing procedures

#### Characterization of MTAC

The maize tassel-based activated carbon (MTAC) was characterized using several analytical techniques to assess its structural, morphological, and surface properties. The specific surface area and porosity of MTAC were determined through Brunauer–Emmett–Teller (BET) analysis, which provided insights into its adsorption potential. Fourier Transform Infrared Spectroscopy (FTIR) was utilized to detect key functional groups hydroxyl (–OH), carboxyl (–COOH), and carbonyl (C = O) on MTAC’s surface, which play a pivotal role in contaminant binding. Scanning Electron Microscopy (SEM) unveiled the adsorbent’s morphological features, including pore distribution and surface roughness, while X-ray Diffraction (XRD) analysis elucidated its crystallinity and structural phases. Collectively, these techniques provided an in-depth evaluation of MTAC’s physicochemical properties, corroborating its effectiveness as a viable adsorbent for industrial wastewater remediation.

#### Collection and composition of industrial wastewater

The synthesized MTAC was evaluated using a batch adsorption setup to treat industrial wastewater obtained from a juice processing factory located in Cairo, Egypt. The characteristics of the raw wastewater are presented in Table 2, which includes key parameters such as alkalinity, total dissolved solids (TDS), chemical oxygen demand (COD), total suspended solids (TSS), pH, and color. As shown in Table 2, the initial COD concentration was approximately 250 mg/L, and the wastewater exhibited a light-yellow color (320 Pt–Co units) with a mildly acidic pH of 5.8, reflecting the organic-rich nature of juice processing effluent.

**Table 2:** Physicochemical characteristics of the industrial wastewater from the juice processing factory (Cairo, Egypt).

Alkalinity	TDS	COD	TSS	pH	Color
mg/L as CaCO_3_	mg/L	mg/L	mg/L	–	Pt–Co units
0	180	250	320	5.8	Light-yellow (320)

#### Analysis of raw and treated water

The pH values of untreated and treated wastewater samples were quantified using an AD1000 pH meter. Total Suspended Solids (TSS) and Total Dissolved Solids (TDS) levels were evaluated following standardized protocols. Chemical Oxygen Demand (COD), a key indicator of organic pollutant concentration, was measured via established analytical methods to gauge contaminant load. All analyses were carried out using high-precision instruments, with pH adjustments made using 0.1 M NaOH or HCl, depending on the experimental conditions. Metal ion concentrations and other parameters were quantified using atomic absorption spectroscopy (AAS) with a ZEEnitu 700P-Analytik Jena-Germany flammatic absorption spectrometer, adhering to standardized analytical protocols. This method ensured precise and reliable detection of contaminants. These analytical procedures provided a comprehensive assessment of the wastewater’s physicochemical characteristics, forming a solid foundation for evaluating the effectiveness of maize tassel-based activated carbon (MTAC) in wastewater treatment.

### Statistical analysis

All adsorption experiments were conducted in triplicate, and the results are presented as mean ± standard deviation to ensure accuracy and reproducibility. Error bars corresponding to standard deviation values have been added to all applicable figures. For comparative analysis, the performance of maize tassel-derived activated carbon (MTAC) and commercial activated carbon (CAC) was statistically evaluated using Minitab® 21.4 software. A two-sample t-test (two-tailed, assuming unequal variances) was applied to determine the significance of differences in adsorption capacity and COD removal between MTAC and CAC. A significance level of α = 0.05 was used, and p-values less than 0.05 were considered statistically significant.

These statistical analyses confirmed that MTAC demonstrated significantly higher adsorption efficiency and COD removal compared to CAC under identical experimental conditions. The use of Minitab ensured precise validation of observed differences and supported the reliability of the comparative findings.

To validate the observed differences between MTAC and commercial activated carbon (CAC), a two-sample t-test was conducted using Minitab software. As shown in Table 3, the differences in both adsorption capacity and COD removal efficiency were found to be statistically significant (*p* < 0.05), confirming the superior performance of MTAC under identical experimental conditions.

**Table 3:** Statistical comparison between MTAC and CAC performance (two-sample t-test using Minitab, α = 0.05).

Performance parameter	MTAC (Mean ± SD)	CAC (Mean ± SD)	*p*-value	Significance (*p* < 0.05)
Adsorption capacity (mg/g)	484.0 ± 8.6	370.0 ± 10.2	0.0004	Significant
COD removal efficiency (%)	96.8 ± 1.1	85.0 ± 1.5	0.0002	Significant

## Results and discussion

### Characterization of activated carbon

#### FTIR spectroscopy

Fourier-transform infrared (FTIR) spectroscopy is a versatile analytical method extensively used to characterize surface functional groups on porous adsorbents like activated carbon. These functional groups critically govern adsorption mechanisms by mediating interactions between the adsorbent and contaminants, thereby dictating pollutant removal efficiency. In the context of biomass-derived activated carbon, FTIR analysis offers valuable insights into the chemical structure and surface chemistry, which directly correlate with its adsorption performance.

The FTIR spectrum of maize tassel-derived activated carbon (MTAC), synthesized at an activation temperature of 800°C, displays distinct absorption bands corresponding to critical surface functional groups. Prominent peaks include carboxyl (–COOH), hydroxyl (–OH), carbonyl (C=O), and aromatic C=C stretching vibrations. These functional groups facilitate enhanced electrostatic and chemical interactions between the adsorbent and pollutant molecules, thus reinforcing the material’s superior adsorption performance in contaminant removal applications.

The presence and intensity of these peaks confirm the successful development of surface functionalities during the carbonization and activation processes. Therefore, FTIR spectroscopy not only confirms the chemical composition of functional groups but also aids in evaluating the material’s efficacy for environmental remediation applications.

A summary of the major functional groups commonly observed in FTIR analysis of activated carbon, along with their associated wavenumbers and vibration types, is presented in Table 4.

**Table 4:** Major FTIR peaks observed in maize tassel-based activated carbon.

Wavenumber (cm⁻^1^)	Functional Group	Vibration Type	Description
~ 3400	–OH (hydroxyl groups)	O–H stretching	Indicative of alcohols, phenols, and adsorbed water
~ 2920	–CH₂/–CH_3_	C–H stretching	Aliphatic chains (possible remnants of biomass)
~ 1700	C=O (carbonyl groups)	C=O stretching	Associated with carboxylic acids, ketones, and aldehydes
~ 1600	C=C (aromatic rings)	C=C stretching	Suggests the presence of aromatic structures formed during carbonization
~ 1400	–COO⁻ (carboxylates)	C–O symmetric stretching	Indicative of carboxylic acids or their salts
~ 1100–1000	C–O–C or C–O	C–O stretching	Often linked to alcohols, ethers, or esters

#### XRD results

The crystalline structure of activated carbon synthesized from maize tassel was analyzed using X-ray diffraction (XRD). The diffraction patterns elucidated microstructural transformations induced during the activation process, providing critical insights into the material’s crystallinity and phase composition. A clear diffraction peak observed at 2θ ≈ 25° is characteristic of the graphitic (002) plane, confirming the formation of an ordered carbon structure in the activated carbon. The broad and diffuse diffraction peaks observed in the low-angle regions reflect diminished crystallinity, a hallmark trait of activated carbon due to its predominantly amorphous structure. These findings align with similar studies where the activation process led to an increase in amorphous carbon content, enhancing the material’s porosity. The observed peak positions and intensities further corroborate the effectiveness of the activation method used in developing a highly porous and structurally modified carbon material. A summary of the main diffraction features and their corresponding interpretations is presented in Table 5.

**Table 5:** Summary of XRD Findings for Maize Tassel-Based Activated Carbon.

2θ Position (°)	Corresponding Plane	Observation	Interpretation
≈ 25°	(002)	Broad diffraction peak	Indicates partial graphitization and formation of layered carbon structures
< 20°	–	Absence or low-intensity peaks	Suggests reduced crystallinity and dominance of amorphous carbon
Broad background hump	–	Wide, low-intensity region across 20°–30°	Characteristic of amorphous carbon with disordered structure
No sharp peaks	–	Lack of crystalline phases	Confirms thermal degradation and structural transformation into amorphous carbon

#### Scanning electron microscopy (SEM)

**Fig. 2:**
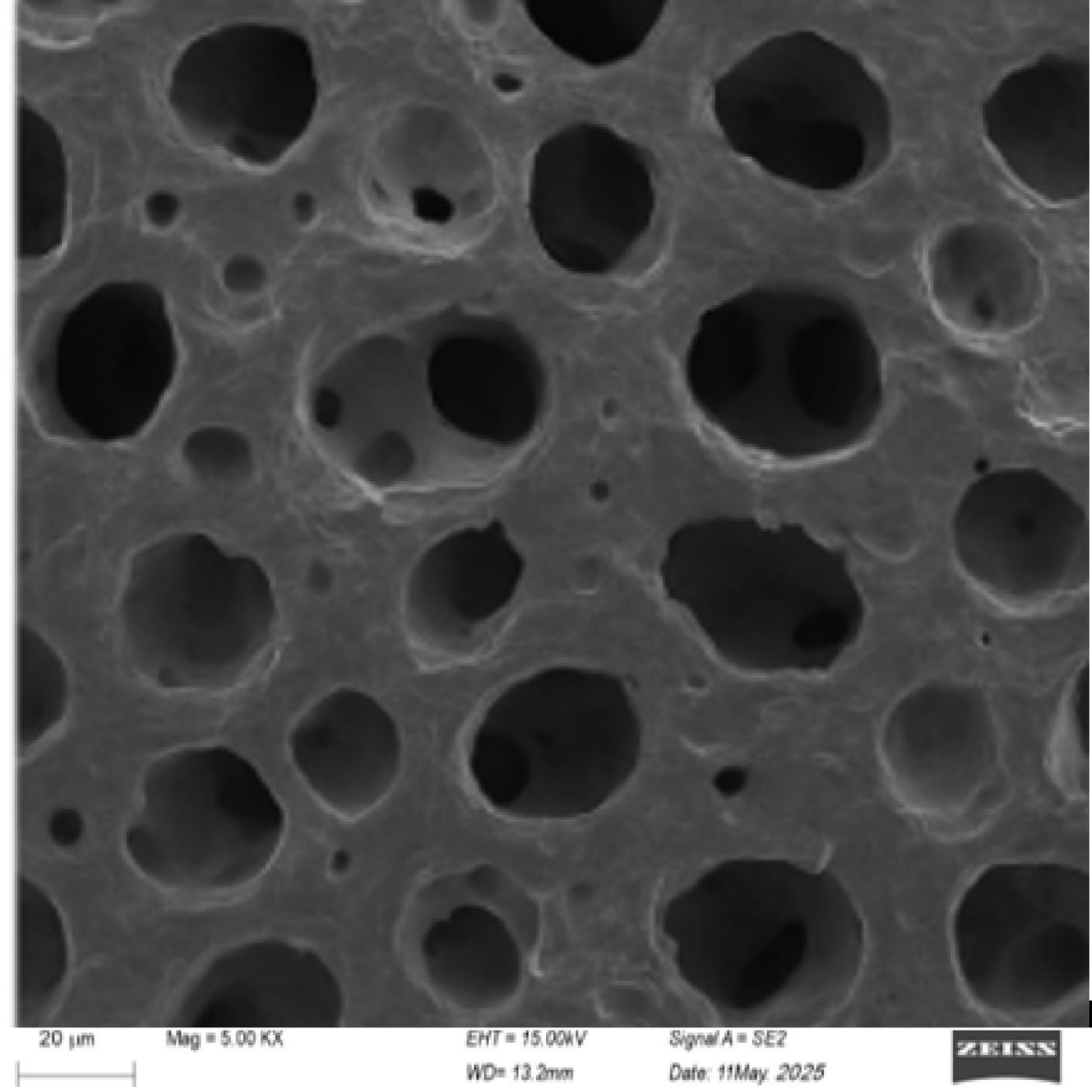
SEM micrographs at 2000×  of magnification for the activated carbon (MTAC).

Scanning electron microscopy (SEM) was employed to analyze the surface morphology of activated carbon synthesized from maize tassels, as depicted in Fig. 2. The SEM images revealed significant changes in the surface structure before and after activation. Raw maize tassels exhibited a smooth and compact surface with minimal porosity, characteristic of the native biomass. However, post-activation at 800°C, the surface morphology of the activated carbon underwent significant structural modification, characterized by the emergence of a highly porous and irregularly rough texture. The presence of well-defined pores and irregular cavities indicates the successful activation and the formation of a high surface area, which is crucial for enhancing adsorption capacity. These structural modifications, as observed under SEM, align with the increase in adsorption performance reported in the FTIR and other characterization studies, confirming that the activation protocol successfully improved the adsorbent’s adsorptive efficiency, underscoring its suitability for contaminant mitigation.

#### BET surface area and pore structure analysis

The surface area, pore volume, and pore diameter of the maize tassel-derived activated carbon (MTAC) were evaluated using nitrogen adsorption desorption isotherms and Brunauer Emmett Teller (BET) analysis. The results revealed that MTAC possessed a BET surface area of 510.4 m^2^/g, a total pore volume of 0.472 cm^3^/g, and an average pore diameter of 3.71 nm (calculated using the BJH method). These values confirm that MTAC exhibits a mesoporous structure, which is highly advantageous for the adsorption of organic pollutants, such as those contributing to chemical oxygen demand (COD) in industrial wastewater. The high surface area and favorable pore characteristics facilitate enhanced mass transfer, greater accessibility of adsorbate molecules to active sites, and improved adsorption efficiency. These findings are consistent with the observed high adsorption capacity (up to 484 mg/g) and support the material’s effectiveness as a low-cost, sustainable adsorbent for wastewater treatment applications. The detailed textural and physicochemical characteristics of MTAC, including BET surface area, total pore volume, and average pore diameter, are summarized in Table 6.

**Table 6:** Textural and physicochemical properties of maize tassel-derived activated carbon (MTAC).

Property	Value	Method/Instrument
BET surface area (m^2^/g)	510.4	BET analysis
Total pore volume (cm^3^/g)	0.472	N₂ adsorption–desorption isotherm (BET)
Average pore diameter (nm)	3.71	BJH method
Functional groups	–OH, –COOH, C=O, C=C	FTIR spectroscopy
Crystallinity phase	Amorphous (002 peak at ~ 25°)	XRD analysis
Surface morphology	Highly porous, rough	SEM imaging

### Batch adsorbent

#### Effect of pH

**Fig. 3:**
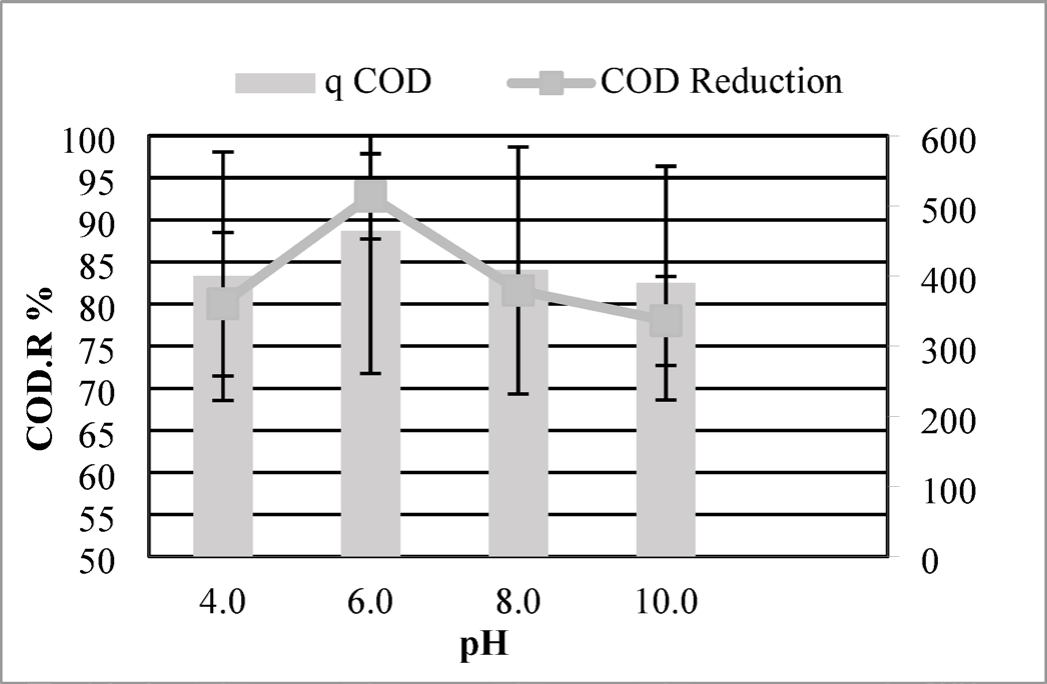
Effect of pH on COD removal efficiency using MTAC: maximum removal observed at pH 6 (96.8%).

The impact of pH (4–10) on the adsorption efficacy and chemical oxygen demand (COD) removal efficiency of maize tassel-derived activated carbon (MTAC) was systematically analyzed. Adsorption capacity (mg/g) was calculated by quantifying the mass of COD adsorbed per gram of MTAC, while COD reduction (%) was derived from the difference between initial and equilibrium COD concentrations in the aqueous phase. The results, shown in Fig. 3, indicate that pH plays a crucial role in the adsorption performance of MTAC.

Maximum adsorption efficiency (464 mg/g) and COD removal (92.8%) occurred at pH 6, suggesting strong electrostatic affinity between MTAC’s functional groups and organic contaminants under slightly acidic conditions. At pH 8, the capacity dropped slightly to 408 mg/g, with a COD reduction of 81.6%. Similarly, at pH 4, although the adsorption capacity was still relatively high (400 mg/g), the COD reduction was only 80%. At pH 10, both adsorption capacity (390 mg/g) and COD reduction (78%) declined, suggesting reduced efficiency in alkaline environments.

These results clearly demonstrate that the adsorption process is highly sensitive to the solution pH, with optimal performance observed at pH 6. The enhanced adsorption under slightly acidic conditions may be attributed to favorable surface charge interactions and increased availability of active functional groups on the MTAC surface for binding organic pollutants.

#### Effect of temperature

**Fig. 4:**
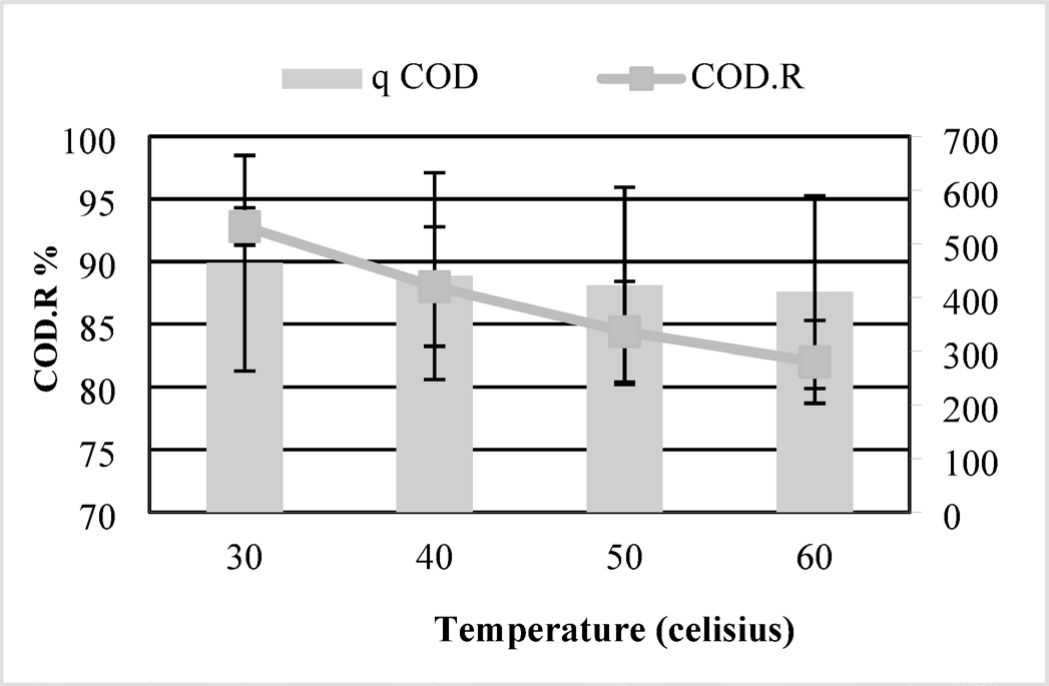
Influence of temperature on COD removal efficiency using MTAC: maximum removal (96.8%) observed at 30 °C, with performance declining at higher temperatures.

Temperature is a critical factor influencing the adsorption process, as it affects the mobility of adsorbate molecules, surface interactions, and the structural behaviour of the adsorbent. To evaluate the effect of temperature on the adsorption performance of maize tassel-derived activated carbon (MTAC), experiments were conducted at 30°C, 40°C, 50°C, and 60°C. Figure 4 illustrates that elevated temperatures led to a progressive reduction in both adsorption capacity and COD removal efficiency.

At a temperature of 30°C, MTAC attained its highest adsorption performance, with a capacity of 464 mg/g and a COD removal efficiency of 92.8%, underscoring the effectiveness of mild thermal conditions for adsorption. When the temperature was elevated to 40 °C, both metrics declined adsorption capacity fell to 440 mg/g, and COD removal decreased to 88% demonstrating reduced efficacy at higher temperatures. Further increases in temperature to 50 °C and 60 °C led to additional reductions in capacity (422 mg/g and 410 mg/g, respectively), accompanied by COD reduction efficiencies of 84.4% and 82%.

These results suggest that the adsorption process is exothermic, as higher temperatures negatively impacted the adsorption performance. The decline in efficiency at elevated temperatures may be attributed to increased molecular motion, which can weaken the physical interactions between adsorbate molecules and active sites on the adsorbent surface, or even cause partial desorption. Thus, 30°C appears to be the optimal temperature for maximum adsorption efficiency, reinforcing the suitability of MTAC for use under ambient or slightly warm conditions in environmental applications.

#### Effect of adsorbent dose

The quantity of adsorbent utilized is a critical determinant in adsorption efficiency, as it directly governs the availability of active binding sites for pollutant sequestration. In this investigation, the influence of maize tassel-derived activated carbon (MTAC) dosage (0.5–3 g/L) on organic contaminant adsorption was systematically evaluated, with initial pollutant concentrations held constant to isolate dose-dependent effects.

**Fig. 5:**
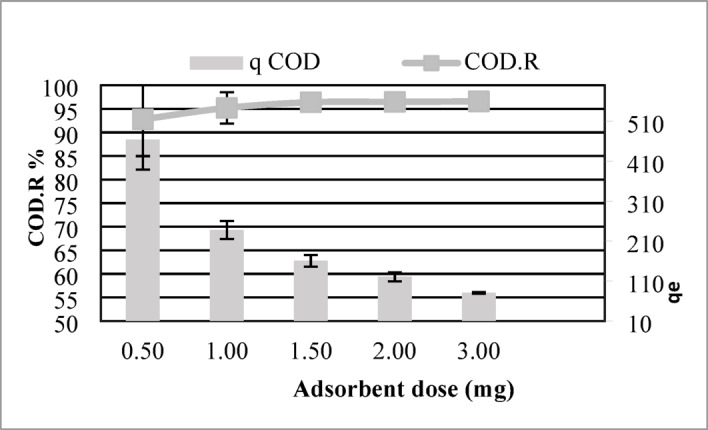
Effect of MTAC dosage on COD removal. Highest efficiency (96.6%) achieved at a dose of 3 g/L.

As shown in Fig. 5, increasing the adsorbent dose resulted in a significant enhancement in COD reduction, reaching 95.2% at 1 g/L, and up to 96.6% at 3 g/L. The enhanced pollutant removal efficiency is driven by the increased availability of adsorption sites due to higher MTAC doses, enabling more effective sequestration of contaminants from the solution. However, a decrease in adsorption capacity (mg/g) was observed as the dose increased. For instance, the adsorption capacity declined from 464 mg/g at 0.5 g/L to 80.5 mg/g at 3 g/L. This inverse relationship occurs because, while more active sites are available at higher doses, the fixed amount of contaminant becomes distributed across a larger mass of adsorbent, resulting in a lower calculated uptake per gram of adsorbent.

These findings highlight that although higher doses improve removal efficiency, the adsorption capacity per unit mass decreases, and therefore, an optimal balance between removal efficiency and material cost must be considered for practical and economic applications.

#### Effect of contact time

Contact time plays a pivotal role in adsorption dynamics, as it directly dictates the period available for interaction between adsorbate molecules and the adsorbent surface. To evaluate its impact, batch experiments were conducted across a range of contact durations (20–60 min), with adsorbent dosage, pH, and temperature maintained at constant levels to isolate temporal effects.

**Fig. 6:**
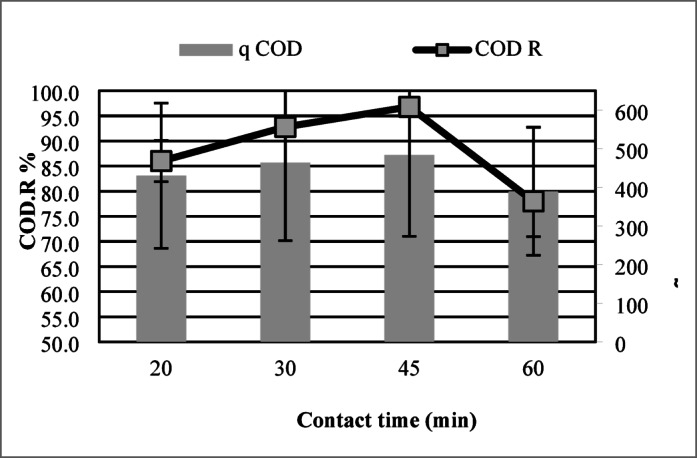
OD removal versus contact time. Maximum adsorption capacity (484 mg/g) and 96.8% removal were reached at 45 min.

Prolonged interaction time significantly improved adsorption efficacy, with Fig. 6 showing a peak capacity of 484 mg/g and 96.8% COD reduction at 45 min. The adsorption process exhibited an initial rapid phase, with capacity escalating from 430 mg/g at 20 min (86% COD removal) to 464 mg/g at 30 min (92.8% COD removal), driven by the high density of accessible active sites on maize tassel-derived activated carbon (MTAC). Beyond this period, the adsorption rate diminished progressively, stabilizing near equilibrium conditions by 45 min.

Interestingly, a slight decline in adsorption capacity was recorded at 60 min (390 mg/g and 78% COD reduction), which may be attributed to possible desorption or redistribution of adsorbed molecules. This behavior suggests that 45 min is the optimal contact time for achieving maximum adsorption efficiency under the experimental conditions.

These findings confirm the typical adsorption profile, characterized by a fast initial uptake followed by a plateau, indicating equilibrium. Therefore, selecting an appropriate contact time is essential to optimize treatment performance and operational efficiency.

#### Effect of agitation intensity (rpm)

Agitation intensity plays a critical role in adsorption kinetics by modulating solute migration from the bulk solution to the adsorbent surface, directly impacting removal efficiency. In this work, batch adsorption trials were conducted at agitation rates of 200–700 rpm, with adsorbent dosage, contact time, pH, and initial pollutant concentration held constant to isolate the influence of mixing dynamics.

**Fig. 7:**
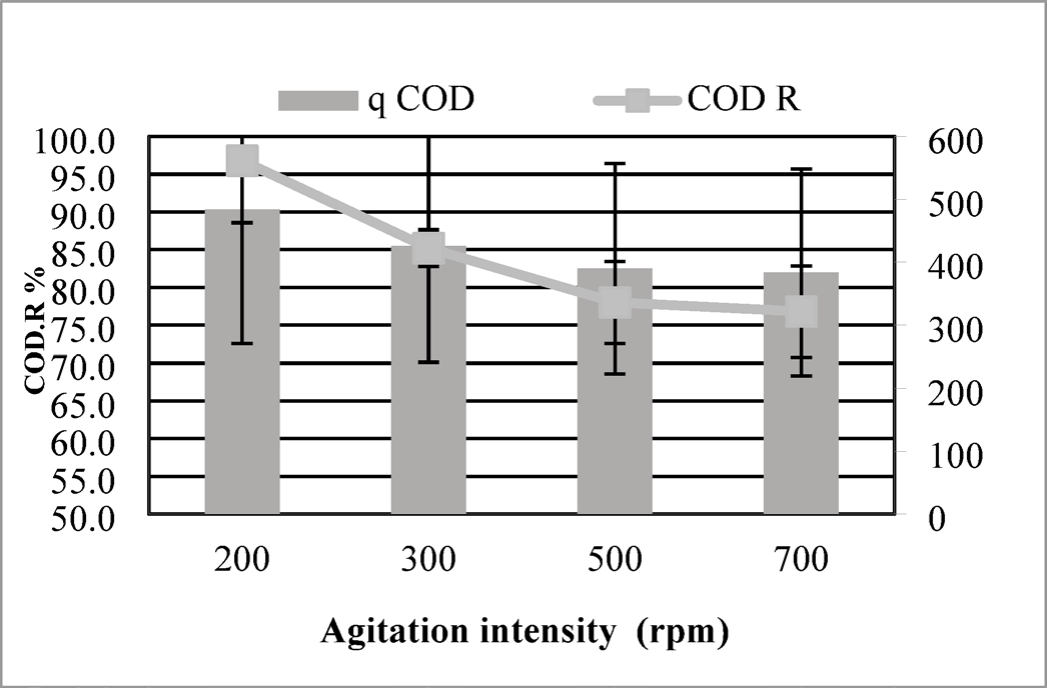
Impact of agitation speed on adsorption performance. Optimum COD removal (96.8%) obtained at 200 rpm.

As shown in Fig. 7, the adsorption capacity and COD reduction initially increased with Agitation intensity, reaching a maximum value of 484 mg/g and 96.8% COD reduction at 200 rpm. This enhancement is attributed to improved mixing and increased interaction between the maize tassel-derived activated carbon (MTAC) and the contaminants in the solution.

Notably, increased mixing rates led to incremental deterioration in adsorption performance. At 300 rpm, the capacity decreased to 426 mg/g with 85.2% COD reduction, and further declined at 500 rpm (390 mg/g, 78%) and 700 rpm (384 mg/g, 76.8%). This reduction can be linked to excessive turbulence at high speeds, which may cause desorption or reduce the effective contact time between adsorbate and adsorbent particles.

These findings indicate that 200 rpm is the optimal Agitation intensity under the tested conditions, as it ensures efficient mass transfer without introducing disruptive turbulence. Selecting an appropriate Agitation intensity is therefore crucial to maximizing adsorption efficiency in practical applications.

### Optimal conditions for batch adsorption

Based on the comprehensive evaluation of various operational parameters, the optimal conditions for maximizing the adsorption of organic contaminants from juice industry wastewater using maize tassel-derived activated carbon (MTAC) were determined.

Optimal adsorption performance was achieved at pH 6, with a capacity of 464 mg/g and 92.8% COD reduction, suggesting that a moderately acidic environment amplifies electrostatic forces and interfacial interactions between the adsorbent and pollutant molecules. Regarding temperature, the optimum was 30°C, where the adsorption capacity remained at its maximum (464 mg/g), and COD removal reached 92.8%. Increasing temperature beyond this point resulted in a gradual decline in performance, suggesting that the adsorption process is exothermic under these conditions.

With respect to adsorbent dose, the best removal efficiency (96.6%) was achieved at 3 g/L, balancing between maximum contaminant removal and material economy. Although increasing the dose improved COD reduction, it caused a decrease in adsorption capacity per gram due to the fixed initial contaminant load.

For contact time, equilibrium was reached at 45 min, where the adsorption capacity peaked at 484 mg/g and COD reduction was 96.8%. Extending the contact time beyond this point did not significantly enhance removal, indicating the establishment of adsorption equilibrium.

Lastly, Agitation intensity had a notable effect, with the optimal performance obtained at 200 rpm, corresponding to an adsorption capacity of 484 mg/g and 96.8% COD reduction. Excessive agitation (≥ 500 rpm) caused a drop in performance, likely due to reduced contact efficiency and desorption effects caused by turbulent flow.

In conclusion, the optimal batch adsorption conditions for MTAC are:

- pH: 6
- Temperature: 30 °C
- Adsorbent dose: 3 g/L
- Contact time: 45 min
- Agitation intensity: 200 rpm

These conditions collectively ensure high adsorption efficiency and provide a foundation for scaling up the process for practical wastewater treatment applications.

Under optimized conditions, MTAC achieved a maximum adsorption capacity of 464 mg/g and COD removal efficiency of 92.8%. The initial COD concentration of the industrial wastewater was 250 mg/L, and after treatment, the residual COD concentration was reduced to approximately 17.8 mg/L. This significant reduction illustrates the effectiveness of MTAC in eliminating organic pollutants and highlights its potential as a cost-effective alternative to commercial activated carbon.

### Comparison with commercial activated carbon

To evaluate the performance of maize tassel-derived activated carbon (MTAC), its adsorption capacity and COD reduction efficiency were compared with those of a commercially available activated carbon (CAC) under identical operating conditions. The experiments were conducted using juice industry wastewater with a COD concentration of approximately 250 mg/L.

**Fig. 8:**
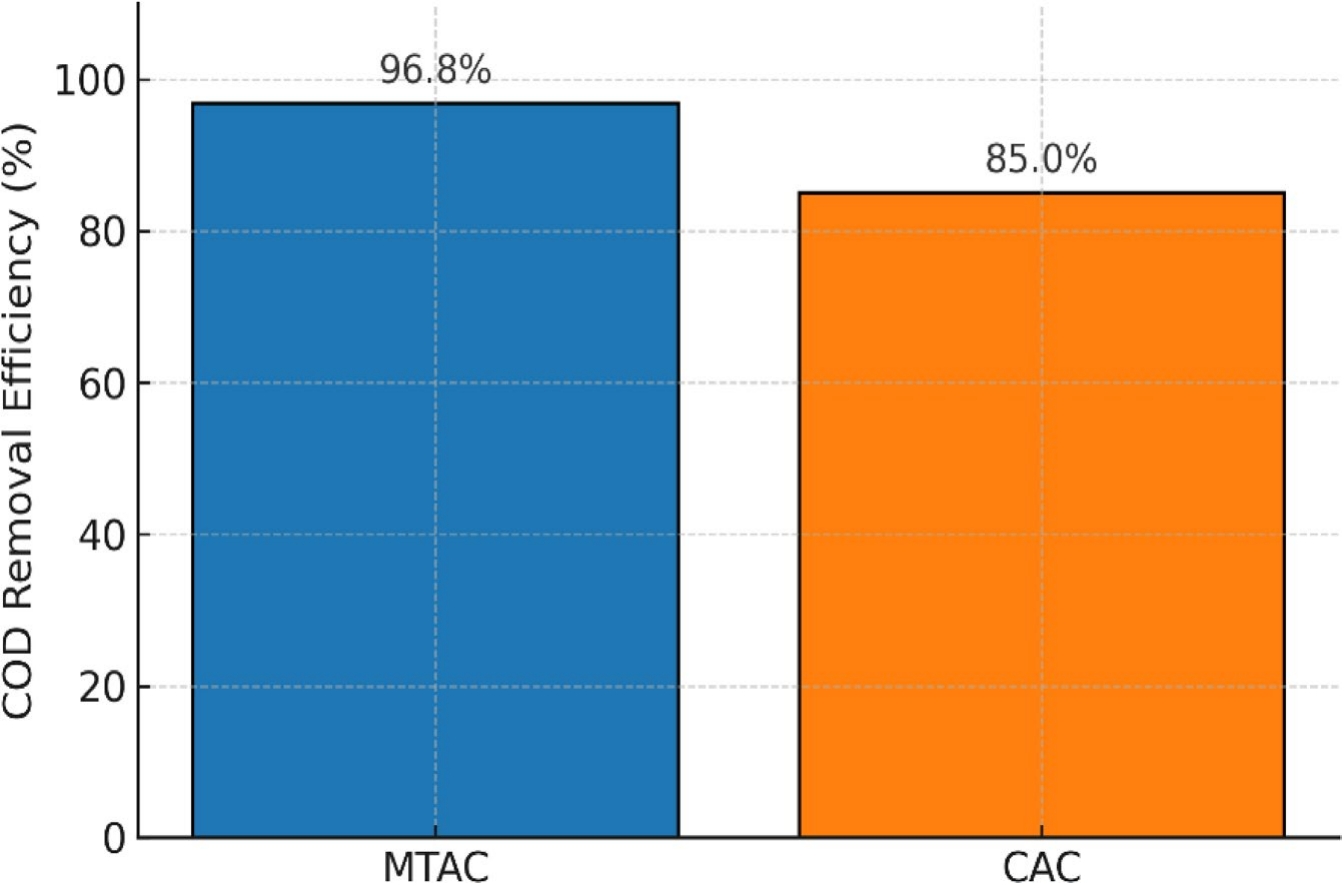
Comparative COD removal efficiency of maize tassel-derived activated carbon (MTAC) and commercial activated carbon (CAC) under identical operating conditions.

Under optimized conditions, MTAC demonstrated significantly enhanced adsorption performance, attaining a peak capacity of 484 mg/g and 96.8% COD removal efficiency. In comparison, commercial activated carbon (CAC) achieved lower efficacy, with a maximum capacity of 370 mg/g and 85% COD reduction under identical experimental settings. This performance comparison is visually presented in Fig. 8, clearly illustrating the superior removal efficiency of MTAC over CAC.

The enhanced performance of MTAC compared to conventional activated carbon stems from its superior structural and chemical properties, including a larger surface area, optimized porosity, and abundant functional groups. These attributes, rigorously confirmed through BET surface area analysis, SEM imaging, and FTIR spectroscopy, facilitate stronger interactions with contaminants, thereby improving adsorption efficiency. Additionally, the MTAC offered the advantage of being cost-effective and derived from agricultural waste, aligning with sustainable waste management and environmental protection practices.

In summary, the comparison clearly indicates that MTAC not only competes favorably with commercial activated carbon but may also offer enhanced performance for COD removal in industrial wastewater treatment, particularly in applications targeting low to moderate organic pollutant loads such as juice processing effluents.

The adsorption capacity of MTAC (484 mg/g) significantly exceeds many previously reported biosorbents for COD removal, reflecting its enhanced surface area (510.4 m^2^/g), mesoporous structure, and abundant functional groups. These attributes were validated through BET, SEM, FTIR, and XRD analyses.

Furthermore, MTAC demonstrated a cost advantage of approximately 64% over CAC, with an estimated production cost of 1.25 USD/kg, offering a promising solution for large-scale industrial applications where cost constraints and sustainability are critical.

By utilizing an underexplored agricultural waste, the study not only introduces a novel material to the field of adsorption science but also contributes to resource-efficient wastewater treatment aligned with SDGs 6 (Clean Water), 12 (Responsible Consumption and Production), and 13 (Climate Action).

### Comparative evaluation of MTAC and CAC

To assess the practical feasibility of maize tassel-derived activated carbon (MTAC) for industrial wastewater treatment, a comparative analysis was conducted with a commercially available activated carbon (CAC). The comparison included key performance indicators such as adsorption capacity, COD removal efficiency, and estimated material cost. The results are summarized in Table 7.

**Table 7:** Comparative performance and cost analysis of MTAC and CAC for COD removal.

Parameter	MTAC	CAC
Adsorption capacity (mg/g)	484	370
COD removal efficiency (%)	96.8	85.0
Estimated cost (USD/kg)	1.25	3.50

Table 7 presents the adsorption capacity of MTAC (484 mg/g) and CAC (370 mg/g) under identical experimental conditions. MTAC also achieved a higher COD removal efficiency (96.8%) compared to CAC (85.0%). From an economic perspective, MTAC demonstrated a substantial cost advantage, with an estimated production cost of 1.25 USD/kg, compared to 3.50 USD/kg for CAC.

**Fig. 9:**
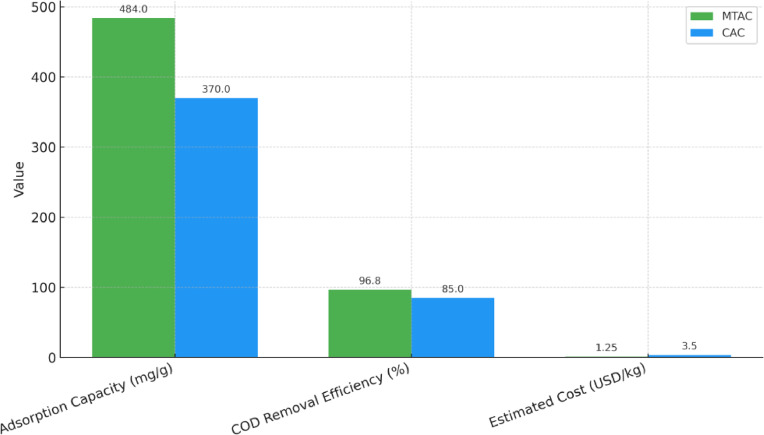
Comparative performance and cost analysis of MTAC and CAC in terms of adsorption capacity, COD removal efficiency, and material cost.

To visualize these differences, a comparison bar chart is provided in Fig. 9, clearly illustrating the superior performance and cost-effectiveness of MTAC. This affirms its potential as a sustainable, low-cost adsorbent for treating industrial effluents, particularly in resource-limited settings.

### Regeneration and reusability study

The reusability of an adsorbent is a crucial factor in evaluating its cost-effectiveness and sustainability for practical wastewater treatment. In this study, the regeneration potential of maize tassel-derived activated carbon (MTAC) was assessed through multiple adsorption–desorption cycles. After each adsorption cycle, the saturated MTAC was subjected to chemical desorption using 0.1 M NaOH solution as the desorbing agent. The procedure involved immersing the used MTAC in NaOH solution for 4 h under agitation (200 rpm), followed by washing with distilled water to neutral pH and drying at 80°C for reuse. This cycle was repeated five times to evaluate the regeneration efficiency. As listed Table 8, and FigureS1 (Supplementary File) the COD removal efficiency decreased gradually over successive cycles but remained above 85% even after the fifth cycle. This decline is attributed to the gradual blockage or degradation of active sites during repeated use.

**Table 8:** Regeneration performance of MTAC over five adsorption–desorption cycles.

Cycle Number	COD Removal Efficiency (%)	Retention Efficiency (%)
1 (Fresh MTAC)	96.8	100.0
2	93.2	96.3
3	89.6	92.6
4	87.4	90.3
5	85.1	87.9

### Cost evaluation

The cost-effectiveness of adsorbents plays a critical role in determining their practical applicability for large-scale wastewater treatment. In this study, a comparative economic assessment was performed between maize tassel-derived activated carbon (MTAC) and commercial activated carbon (CAC), focusing on two key indicators: unit cost (USD/kg) and estimated treatment cost per cubic meter of water (USD/m^3^).

#### Raw material and production cost of MTAC

MTAC was synthesized from maize tassels, an abundant agricultural byproduct, which significantly lowers raw material costs. The production process involved chemical activation using phosphoric acid, carbonization, washing, and drying. Based on laboratory-scale cost calculations and literature values, the estimated cost of MTAC production ranges from $0.50 to $0.70 per kg, depending on energy and chemical use.

In contrast, commercially available activated carbon (CAC) has a market price ranging from $2.50 to $3.00 per kg, reflecting the cost of raw material procurement, processing, quality control, and distribution.

#### Treatment cost per cubic meter of wastewater

To provide a practical cost comparison, we calculated the adsorbent dose required per m^3^ of water based on the experimental optimal dosage of 3 g/L, equivalent to 3 kg/m^3^ of wastewater treated.

The cost per cubic meter was then estimated as:

- MTAC:3kg*$0.5–0.7/Kg = $1.50–2.10/m^3^
- CAC: 3kg*$2.5–3.00/Kg = $7.50–9.00/m^3^

However, when accounting for adsorption performance, MTAC demonstrated higher COD removal (96.8%) than CAC (85.0%). Therefore, the effective cost per unit of COD removed further favors MTAC.

Table 9 presents the cost comparison between MTAC and CAC.

**Table 9:** Comparative cost and performance of MTAC vs. commercial activated carbon.

Adsorbent	Estimated Cost (USD/kg)	COD Removal Efficiency (%)	Cost per m^3^ Treated Water (USD)
MTAC	0.50–0.70	96.8	1.50–2.10
CAC	2.50–3.00	85.0	7.50–9.00

These results confirm that MTAC offers a substantial economic advantage over CAC, reducing both material costs and the cost per unit volume of treated water. Combined with its high adsorption capacity and reusability, MTAC is a cost-effective and sustainable alternative for industrial wastewater treatment.

### Model studies

At the optimum pH (6) and an MTAC dose of 3 g/L, the adsorption of COD was evaluated over contact times ranging from 20 to 60 min. To comprehensively interpret the adsorption behavior, equilibrium, kinetic, and thermodynamic models were employed. The equilibrium data were analyzed using the Langmuir and Freundlich isotherm models to describe the adsorption capacity and surface characteristics of MTAC. Adsorption kinetics were examined using the pseudo-first-order and pseudo-second-order models to identify the dominant adsorption mechanism and rate-controlling steps. In addition, thermodynamic parameters were evaluated to determine the feasibility, spontaneity, and nature (endothermic or exothermic) of the COD adsorption process. Collectively, these models provided a detailed understanding of the interaction between COD molecules and MTAC under batch conditions.

#### Isothermal models

Adsorption isotherms describe the equilibrium relationship between the amount of adsorbate retained on the adsorbent and its concentration in solution. In this study, two widely used isotherm models Langmuir and Freundlich were applied to fit the experimental equilibrium data for COD adsorption onto MTAC as shown in Figures S2 to S5 (Supplementary File).

##### Langmuir isotherm

The Langmuir isotherm assumes monolayer adsorption on a homogeneous surface with finite identical sites. The results are summarized in Table 10. The correlation coefficient (R^2^ = 0.996) suggests that the Langmuir model effectively describes the equilibrium adsorption of COD onto MTAC. The maximum adsorption capacity (q_o_) was found to be 484 mg/g, and the Langmuir constant (K_L_) was 0.25 L/mg, indicating a strong affinity between COD molecules and the MTAC surface. The dimensionless separation factor (R_L_) was calculated using the equation:

3 $${R}_{L}=\frac{1}{1+{K}_{L}{C}_{o}}$$

with an initial concentration C_0_ = 250 mg/L, R_L_ was determined to be 0.015, which lies between 0 and 1 signifying that the adsorption process was favorable. These findings suggest that COD molecules were primarily adsorbed onto uniformly distributed active functional groups on the MTAC surface in a monolayer fashion. The high adsorption capacity further demonstrates the effectiveness of MTAC as a sustainable and efficient biosorbent for industrial wastewater treatment.

**Table 10:** Langmuir Isotherm Constants for COD Adsorption Using MTAC.

Pollutant	Langmuir model	Plotting	q_o_ (mg·g⁻¹)	K_L_ (L·mg⁻¹)	R_L_	R^2^
COD	Nonlinear:${q}_{e }={q}_{o}\frac{{K}_{L}{C}_{e}}{1+{K}_{L}{C}_{e}}$ (4)	${q}_{e }vs {C}_{e}$	484	0.25	0.015	0.999
	Linear:${\text{ Ce}}/{\text{q}}_{e} = 1/{\text{q}}_{{\text{o}}} {\text{K}}_{L} + {\text{Ce}}/{\text{q}}_{{\text{o}}}$ (5)	$\text{Ce}/{q}_{e} vs {C}_{e}$	460.76	0.23	0.017	0.996

##### Freundlich isotherm

The Freundlich model explains adsorption on a heterogeneous surface with sites of varied affinities and multilayer adsorption. As shown in Table 11, the Freundlich constant (K_F_) was calculated as 78.50 (mg/g)(L/mg)^1^⁄ⁿ and the heterogeneity factor (n) was 2.23, with an R^2^ value of 0.995. These results indicate strong adsorption affinity and surface heterogeneity of MTAC. The 1/n value, calculated as approximately 0.448, falls between 0 and 1, confirming that the adsorption process is favorable and occurs over a heterogeneous surface. This also supports the possibility of multilayer formation during COD adsorption onto MTAC. However, the linear form of the Freundlich model showed a much lower goodness-of-fit (R^2^ = 0.270), with K_F_ = 74.32 and n = 1.85, suggesting some inconsistency in model fitting across methods. Compared to the Langmuir model, which achieved a better overall fit (R^2^ = 0.999), the Freundlich model is slightly less suitable for describing the equilibrium behavior. Thus, monolayer adsorption remains the dominant mechanism, though surface heterogeneity may still play a contributory role.

**Table 11:** Freundlich Isotherm Constants for COD Adsorption Using MTAC.

Pollutant	Freundlich model	Plotting	KF ((mg/g)/(mg/L)ⁿ)	n	R^2^
**COD**	Nonlinear: ${q}_{e}={K}_{f}{C}_{e}^{1/n}$ (6)	${q}_{e } vs {C}_{e}$	78.50	2.23	0.995
	Linear:$\text{ln}{q}_{e}=\text{ln}{K}_{F}{+\frac{1}{n}\text{ln}C}_{e}$ (7)	$\text{ln}{q}_{e} vs {\text{ln}C}_{e}$	74.32	1.85	0.270

#### Kinetic models

To describe the COD adsorption behavior over time, kinetic studies were conducted using both pseudo-first-order and pseudo-second-order models. These models help identify the controlling mechanism of adsorption whether physical or chemical in nature as shown in Figures S6 to S9 (Supplementary File).

##### Pseudo-first-order kinetic model

The pseudo-first-order kinetic model assumes that the rate of adsorption is directly proportional to the number of available adsorption sites. However, as summarized in Table 12, the model showed a low correlation coefficient in both nonlinear and linear forms for COD adsorption onto MTAC. The nonlinear form resulted in a rate constant k_1_ = 0.146 min^−1^, an equilibrium adsorption capacity q_e_ = 55.74 mg·g⁻^1^, and a very low coefficient of determination R^2^ = 0.298, indicating poor agreement with the experimental data. Similarly, the linear form of the model yielded k_1_ = 0.042, q_e_ = 33.60 mg·g⁻^1^, and a moderate fit with R^2^ = 0.712, still insufficient to describe the adsorption kinetics accurately. These results suggest that the pseudo-first-order model does not adequately represent the adsorption mechanism of COD onto MTAC, as the predicted q_e_ ​values do not align well with experimental values and the overall fit is weak. Therefore, this model is unsuitable for describing the adsorption behavior in this system.

**Table 12:** Pseudo-First-Order Kinetic Model Parameters for COD Adsorption.

Pollutant	The pseudo-first-order model	Plotting	k₁ (min⁻^1^)	qₑ (mg·g⁻^1^)	R^2^
COD	Nonlinear: ${q}_{t}={q}_{e} (1-{e}^{{-k}_{1}t})$ (8)	${q}_{t } vs t$	0.146	55.74	0.298
	Linear:$\text{log}\left({q}_{e}-{q}_{t}\right)=\text{log}\left({q}_{e}\right)-{k}_{1}t/\text{ln}10$ (9)	$\text{log}\left({q}_{e}-{q}_{t}\right) vs t$	0.042	33.60	0.712

##### Pseudo-second-order kinetic model

The pseudo-second-order model assumes that chemisorption is the rate-limiting step, involving the sharing or exchange of electrons between the adsorbate and the adsorbent surface. As summarized in Table 13, this model provided an excellent fit to the experimental data for COD adsorption onto MTAC. In the nonlinear form, the model yielded a rate constant k_2_ = 0.0031 g·mg⁻^1^·min⁻^1^, an equilibrium adsorption capacity q_e_ = 61.35 mg·g⁻^1^, and a high coefficient of determination R_2_ = 0.998, indicating strong agreement between predicted and experimental values. Similarly, the linear form of the model produced a rate constant k_2_ = 0.0028 g·mg⁻^1^·min⁻^1^, q_e_ = 58.74 mg·g⁻^1^, and R_2_ = 0.987, also demonstrating an excellent correlation. These results confirm that the adsorption kinetics of COD onto MTAC are best described by the pseudo-second-order model, implying that the dominant mechanism is chemisorption involving strong chemical interactions between the organic pollutants and the functional groups on the MTAC surface.

**Table 13:** Pseudo-Second-Order Kinetic Model Parameters for COD Adsorption.

Pollutant	The pseudo-second-order model	Plotting	k₂ (g·mg⁻^1^·min⁻^1^)	qₑ (mg·g⁻^1^)	R^2^
**COD**	Nonlinear: ${q}_{t}={k}_{2}{q}_{e}^{2}t/(1+{k}_{2}{q}_{e}t)$ (10)	${q}_{t } vs t$	0.0031	61.35	0.998
	Linear:$t/{q}_{t}=1/{(k}_{2}{q}_{e}^{2})+t/{q}_{e}$ (11)	$t/{q}_{t} vs t$	0.0028	58.74	0.987

#### Thermodynamic models

To further elucidate the effect of temperature on the adsorption of COD onto maize tassel-derived activated carbon (MTAC), a thermodynamic investigation was conducted. Thermodynamic parameters including Gibbs free energy change (ΔG°), enthalpy change (ΔH°), and entropy change (ΔS°) were evaluated to substantiate the temperature-dependent adsorption behavior and to confirm the exothermic nature of the process. The equilibrium distribution coefficient (${\text{K}}_{\text{e}}$) was determined using the ratio of equilibrium adsorption capacity (q_e_​, mg/g) to the equilibrium concentration in solution (Ce​, mg/L), expressed as:

$${\text{K}}_{{\text{e}}} = {\text{q}}_{{\text{e}}} /{\text{C}}_{{\text{e}}}                                                 $$

The standard Gibbs free energy was calculated according to:

$$\Delta \text{G}=-\text{RT ln}{\text{K}}_{\text{e}}$$

while the enthalpy and entropy changes were obtained from the van’t Hoff equation:

$${\text{lnK}}_{\text{e}}=\frac{\Delta \text{S}}{\text{R}}-\frac{\Delta \text{H}}{\text{RT}}$$

where R is the universal gas constant (8.314 J·mol⁻^1^·K⁻^1^) and T is the absolute temperature (K). By plotting ${\text{lnK}}_{\text{e}}$​ ​ against 1/T, ΔH° and ΔS° were determined from the slope and intercept, respectively. Both nonlinear fitting (direct regression of experimental K_e_) and linear van’t Hoff transformation were applied to enhance accuracy and validation.

The calculated thermodynamic parameters are summarized in Table 14, while the linear fit is illustrated in Figure S10 (Supplementary File) and the nonlinear van’t Hoff plot in Figure S11 (Supplementary File). The negative values of ΔG° at all investigated temperatures (− 0.64, − 0.66, − 0.70, and − 0.76 kJ·mol⁻^1^ at 303, 313, 323, and 333 K, respectively) confirm that COD adsorption onto MTAC occurs spontaneously. The enthalpy change (ΔH° =  − 0.523 kJ·mol⁻^1^) indicates that the process is exothermic, consistent with the observed decline in K_e_ with increasing temperature. Moreover, the small positive entropy change (ΔS° = 0.003 kJ·mol⁻^1^·K⁻^1^) suggests a slight increase in randomness at the solid–liquid interface, possibly due to partial desolvation of organic molecules during adsorption. These findings validate the experimental data and demonstrate that COD removal by MTAC is a spontaneous and mildly exothermic process, governed primarily by physical adsorption interactions.

**Table 14:** Thermodynamic parameters for COD adsorption onto MTAC.

Pollutant	The thermodynamic model	Temp (K)	K_e_ model	ΔG(KJ/mol)	ΔH(KJ/mol)	ΔS(KJ/mol)	R^2^
COD	${\text{lnK}}_{\text{e}}=\frac{\Delta \text{S}}{\text{R}}-\frac{\Delta \text{H}}{\text{RT}}$ Nonlinear: $\Delta \text{G}=-\text{RT ln}{\text{K}}_{\text{e}}$	303	12.890	-0.640	-0.523	0.003	0.998
	${\text{lnK}}_{\text{e}}=\frac{\Delta \text{S}}{\text{R}}-\frac{\Delta \text{H}}{\text{RT}}$ Linear: $\Delta \text{G}=-\text{RT ln}{\text{K}}_{\text{e}}$	303	12.890	-0.640	-0.523	0.003	0.997

## Conclusions

The experimental findings and analytical deliberations yield the following key conclusions:

- FTIR analysis verified the existence of functional moieties (–OH, –COOH, C = O, and C = C), which improve pollutant adsorption by fostering molecular-level interactions between the adsorbent and contaminants.
- These functional groups indicate successful surface modification during dual-stage protocol: carbonization followed by activation at 800 °C.
- XRD analysis revealed a broad peak at 2θ ≈ 25°, indicating partial graphitization and the presence of layered carbon structures.
- Collectively, FTIR, XRD, and SEM analyses confirm that maize tassel-derived activated carbon (MTAC) exhibits optimal surface functionality, pore architecture, and structural integrity, solidifying its role as an eco-friendly, economical, and efficient adsorbent for environmental remediation.
- Activated carbon synthesized from maize tassels (MTAC) demonstrated high efficacy as a renewable and economical adsorbent for eliminating organic contaminants, particularly chemical oxygen demand (COD), from wastewater in the juice production sector.
- The optimal operational conditions for MTAC were determined to be a pH of 6, temperature of 30°C, adsorbent dosage of 3 g/L, contact duration of 45 min, and agitation speed of 200 rpm. Under these optimized settings, MTAC exhibited a maximum adsorption capacity of 484 mg/g and attained a COD reduction efficiency of 96.8%, highlighting its strong adsorption potential for organic pollutants.
- MTAC demonstrated enhanced adsorption performance (484 mg/g, 96.8% COD removal) compared to CAC (370 mg/g, 85%), attributed to its higher porosity, surface area, and eco-friendly production from agricultural waste.
- The exceptional adsorption performance of MTAC is attributed to its increased surface area, extensive porous architecture, and diverse surface functional groups, as evidenced by BET, SEM, and FTIR characterization analyses.
- MTAC is a low-cost, renewable material derived from agricultural waste, promoting environmental sustainability and resource valorization.
- The results confirm that MTAC maintains high performance even after multiple cycles, indicating its robustness and potential for reuse in industrial-scale wastewater treatment. The relatively minor decrease in efficiency supports the use of MTAC as a low-cost, sustainable, and regenerable adsorbent, aligning with circular economy and green engineering principles.
- This study highlights the potential of maize tassel–based activated carbon (MTAC) as a novel, cost-effective, and environmentally sustainable adsorbent for industrial wastewater treatment. The material’s superior performance, combined with its low production cost and underutilized origin, underscores its practical value and contribution to circular economy strategies.
- Adsorption equilibrium data were best described by the Langmuir isotherm model, indicating monolayer adsorption on a homogeneous surface.
- The Langmuir model showed a high coefficient of determination (R^2^ = 0.999), confirming excellent model fit.
- The Freundlich isotherm model also showed a good fit, with a Freundlich constant n = 2.23, which is greater than 1, suggesting favorable and multilayer adsorption behavior on heterogeneous surfaces.
- Thermodynamic analysis confirmed that COD adsorption onto MTAC is spontaneous (negative ΔG°), exothermic (ΔH° =  − 0.523 kJ·mol⁻^1^) and accompanied by a slight increase in interfacial randomness (positive ΔS°).
- These results confirm that MTAC has a high affinity and capacity for organic pollutant adsorption, with performance superior to that of commercial activated carbon.

## Supplementary Information

Below is the link to the electronic supplementary material.

Supplementary Material 1

## Data Availability

All data generated or analysed during this study are included in this published article and its supplementary information files.
