# Supplementary material for: Performance of maize tassel activated carbon for COD removal from industrial wastewater under optimized conditions
Source: Sci Rep. 2025 Nov 18;15:40378. doi: 10.1038/s41598-025-24902-y (PMC12627661; doi:10.1038/s41598-025-24902-y)
Supplement: Supplementary file 1 — Supplementary Material 1 [file 41598_2025_24902_MOESM1_ESM.docx]

**Supplementary Data**


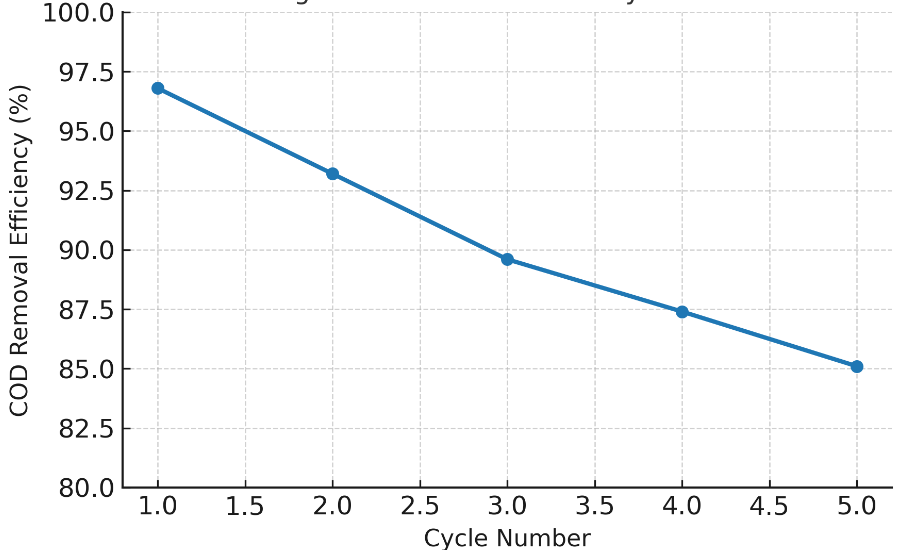


**Figure 1.** Regeneration and reusability performance of MTAC over five consecutive adsorption–desorption cycles for COD removal

**Figure 2.** Adsorption on AgNPs/GO/Chitosan in Langmuir isotherms of Cr ions by linear analysis

**Figure 3.** Adsorption on AgNPs/GO/Chitosan in Langmuir isotherms of Fe ions by Nonlinear analysis

**Figure 4.** Adsorption on AgNPs/GO/Chitosan in Freundlich isotherms of Cr ions by linear analysis

**Figure 5.** Adsorption on AgNPs/GO/Chitosan in Freundlich isotherms of Fe ions by Nonlinear analysis.

**Figure 6.** Adsorption on AgNPs/GO/Chitosan in pseudo-first-order of Cr ions by linear analysis

**Figure 7.** Adsorption on AgNPs/GO/Chitosan in pseudo-first-order of Fe ions by Nonlinear analysis.

**Figure 8.** Adsorption on AgNPs/GO/Chitosan in pseudo-second-order of Cr ions by linear analysis,

**Figure 9.** Adsorption on AgNPs/GO/Chitosan in pseudo-second-order of Fe ions by Nonlinear analysis.

**Figure 10.** Linear van’t Hoff plot for COD adsorption onto MTAC,

**Figure 11.** Nonlinear van’t Hoff plot for COD adsorption onto MTAC.
